# Spring Based Connection of External Wires to a Thin Film Temperature Sensor Integrated Inside a Solid Oxide Fuel Cell

**DOI:** 10.1038/s41598-019-39518-2

**Published:** 2019-02-15

**Authors:** Erdogan Guk, Vijay Venkatesan, Yunus Sayan, Lisa Jackson, Jung-Sik Kim

**Affiliations:** 1 Aeronautical & Automotive Engineering Department, Loughborough University, Loughborough, LE11 3TU United Kingdom; 2 Bozok Üniversitesi Mühendislik-Mimarlık Fakültesi Erdoğan AKDAĞ Kampüsü Atatürk Yolu 7. km, Yozgat, Turkey

## Abstract

Thermal management of SOFCs (solid oxide fuel cell) is important for helping to minimise high temperature-related performance losses and maximising cell/stack lifetime. Thin film sensor technology is proposed as an excellent candidate to measure the cell temperature during operation due to its negligible mass, minimal disturbance to normal operation and higher temporal and spatial resolutions. However, the effective application of such sensors in SOFC systems is a challenging endeavour and predicated on incorporating the external wire attachments to complete the electrical circuit. This is because of the high sensitivity of SOFC materials to any interference to operation, limited available space and harsh operating conditions. In this paper, a new concept of packaging external wire attachments to the thin film sensor is described to enable the integration of the sensor in the SOFC system. Temperature measurements have been monitored under OCV and operating condition with the thin film sensor directly from SOFC cathode surface via proposed spring-based wire connection, from room temperature to SOFC operating temperature. The impact of the parameters including contact resistance (Rc) between sensor pads and attached wire on monitored temperature has also been analysed with the contribution of conductive paste. High temporal and spatial resolutions have been obtained with the implemented sensor.

## Introduction

Solid oxide fuel cells (SOFC) have been considered as a promising technology with their high efficiency (>50%), reaction kinetics, fuel flexibility and high power output^1,2^. There is a possibility to further increase its efficiency up to 70% by integration with other energy generating systems such as gas turbines or steam turbines in which the exhausted heat from SOFC systems can be utilized/recovered^3^.

However, high system cost and ensuring reliability are the main challenges to be overcome for fully commercialising SOFC technologies^4–7^. The most important parameter directly related with high cost and reliability is the high operating temperature (800–1000 °C) of SOFC^6^. As a result of high temperature, thermal stresses across the cell materials are induced due to different coefficient of thermal expansion (CTE) of the SOFC components, leading to micro-scale cracking, resulting in cell (and possibly system) failure^8–10^. Therefore, understanding the temperature characteristics of SOFC is a key point to overcome high temperature-related issues.

Due to harsh operating environments of a working cell, it is difficult to measure the temperature of operating SOFCs experimentally^11,12^. Thus, despite a great number of numerical analysis-related work^13–16^, only a limited number of experimental studies are available^17^. For example, a detailed numerical simulation tool for a planar SOFC was developed by Wang *et al*.^18^ and applied to identify the effect of operating conditions and anode structure on the cell performance. This study also confirms that co-flow type configuration leads to a more uniform temperature profile. However, work by Aydin has claimed^19^ that further validation is required for the SOFC numerical analyses, by comparison with experimental results; this is held especially for those numerical studies conducted by considering the conventional I–V curve.

Thermocouples (TCs) have been a widely applied measurement technique for experimental temperature measurement from an operating SOFC^20^. The main challenges in conducting temperature measurements with TCs is the impinging of limited available space and inducing of disturbance to the operating environment of an SOFC system^21^. Therefore, the measurements from TCs in SOFCs is generally obtained from gas flow channels with an allowed adjacency to the cell surface^17^. With respect to this arrangement, there is a disagreement about the reliability of the measured temperature as to whether it represents the actual cell surface temperature^22^. Thus, obtaining measurements directly from SOFCs’ electrode surface is important with minimum disturbance to the larger working environment of SOFC system. A thin film- based sensor architecture is realised as a potential application for SOFC temperature sensing due to their advantageous features that include minimal intrusion to the normal working of the cell and comprising of mechanically self-supporting components not requiring too much machining^23^. Furthermore, it is capable of higher temporal and spatial resolutions. The temporal resolution is appreciably high with the thin film structured TCs from the reduced thermal inertia of the layers, which translates to providing faster signal response compared to conventional TCs^24^. Additionally, the spatial resolution is also higher due to direct contact between sensing system and the location that the temperature to be measured^25^. The persisting challenge is to ensure that, as this is still fundamentally an analogue electrical method, the external wiring and connections, which are critical for the signal acquisition, are made reliable, conductive and robust.

In this study, a technique for wire attachment, designated as spring-based connection (SBC), has been investigated at SOFC operating temperature. The cell holder which accommodates SOFC planar cell and its main components in relation with the wire connection method is elaborated. The external wire connection mechanism to the sputtered thin film sensor pads is also described. Temperature measurement on the cathode surface has been carried out with the proposed method and the parameters affecting the monitored reading were also identified. Temperature variation of the SOFC cell under different operating conditions is monitored. The wider intent is to extend/replicate the design provisions for these external connections at a stack level to allow sensory techniques to be more easily applicable for SOFCs that eventually can help to (i) understand the degradation issues of SOFC systems deeply and (ii) provide an integrated platform for SOFC lifecycle monitoring and diagnostics that more accurately includes temperature monitoring, at both a cell- and stack-wide level.

## Results and Discussions

### Results for SBC without silver paste

**Figure 1:**
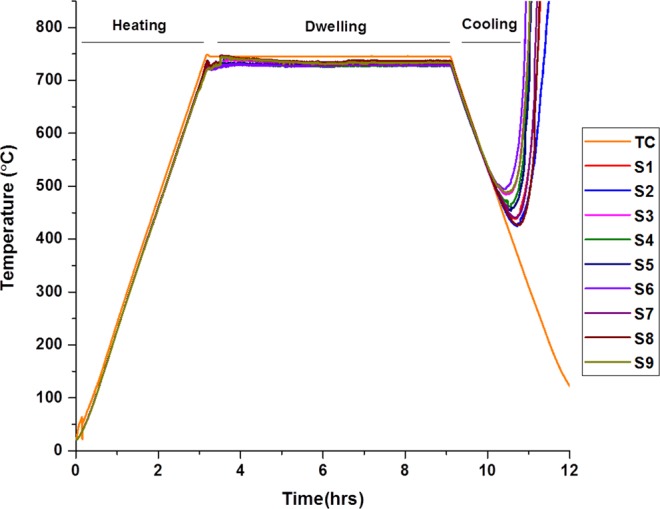
Temperature measurements with spring based connection without silver full experiment.

Figure 1 shows the temperature measurement obtained by sensor sensing points (SSPs) and TC from the experiment with the SBC without any silver paste.

**Figure 2:**
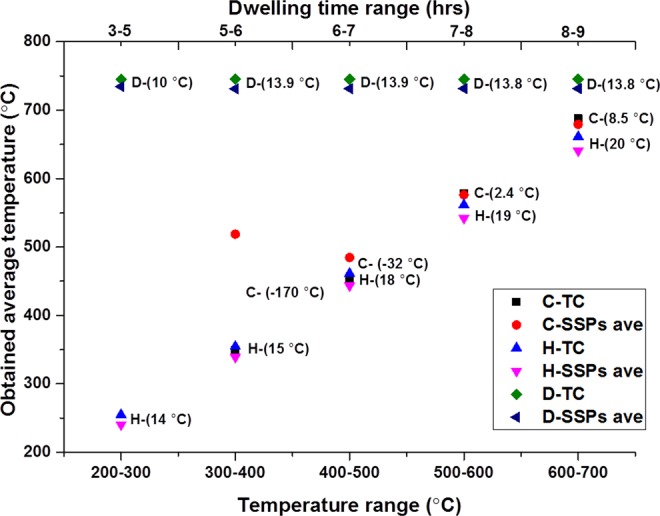
SSPs and TC average temperature from the given temperature range and the differences between them.

Nine readings from the SSPs (S1–S9) and a reading from the commercial TC are plotted. There is comparatively good agreement with the reading from sensor and TC during heating and dwelling segments compared to cooling segment. TC provides higher reading than the SSPs throughout the experiment till failure. Figure 2 demonstrates TC and SSPs-ave (average over S1–S9) average temperature reading from the given range as well as the temperature difference (TC–SSPs-ave) of TC and SSPs at the averaged reading for heating, cooling, and dwelling segments. There is 14 °C temperature difference observed between TC and SSPs-ave whilst the average cell temperature was at about 250 °C during heating segment (H-TC, and H-SSPs ave).

This is primarily presumed to be due to higher thermal inertia of the bulk cell than the air atmosphere in the furnace. Thermal inertia, *I*, can be described as a measurement of material’s resistance to their surrounding temperature changes^26^. It can be altered depending on the materials density (*p*), specific heat capacity (*c*) and thermal conductivity (*k*) with the given relationship between them in “(1)”.

1 $$I=\surd (ckp)$$

with respect to the relationship (1), it is reasonable to obtain higher readings from TC which was located at one of the gas flow channels 2–3 mm above the cell cathode that measures the furnace atmosphere temperature while cell surface temperature is measured by the SSPs. However the difference is not constant and increases gradually with the increase in temperature while furnace heating rate and cell thermal inertia are constant (Fig. 2).

It points to another source of difference: that the difference in the resistance properties of the TC and SSPs is possibly due to structural differences of the thin film sensor which has less initial resistance (Table 1).

**Table 1:** Initial resistance of SSPs and TC.

Parameters	Resistance (Ω) before experiment at 20 °C
SSPs-ave (S1–S9)	25
TC	52

Importantly, the variation in the temperature difference within TC and SSPs-ave rises from 14 °C to 20 °C with the increase in obtained average temperature from 250 °C to 750 °C (Fig. 2). It indicates that there is a distinct alteration in the resistances of TC and SSPs-ave resulting in more changes in the temperature reading than the others even with similar rate of changes occurring in surrounding temperature. The properties of the electrical resistance are given by “(2)”.

2 $$R=l\rho /A$$

where, “*R”* is resistance, “*l”* is length, “*ρ*” is resistivity and “*A”* is the cross section area. As inferred from (2) the increase in resistivity which increases with increasing temperature leads increase in resistance *“R”*. And the increase in resistance leads to an increase in voltage. However, it has small or negligible impact on the variation in temperature as the current in thermocouples is negligibly small and controlled by high impedance amplifier(s). Additionally, according to the Seebeck theory, voltage is only dependant on the parameters given below in (3):

3 $${V}_{emf}={\int }_{T0}^{T1}({S}_{A}-(\,-\,{S}_{C}))dT$$

where “*V*_*emf*_” is Seebeck potential, “*S*_*A*_” and “*S*_*C*_” are the Seebeck coefficients of the two thermoelement materials alumel and chromel, respectively, while T0 is the temperature at the terminal and T1 is the temperature at the junction.

It is apparent that the higher the Seebeck coefficient, the higher the corresponding Seebeck potential resulting in higher temperature reading (3). Additionally, the increase in thermal resistance (R_th_) of the thermoelements by operating temperature leads to an increase in Seebeck coefficient^27^. However, the thermoelements used for both TC and SSPs are the same (alumel and chromel), along with their wire sizes being almost similar in length (1 meter) and cross sectional dimension (Φ 0.25 mm). The only difference is the thin film sensor pattern itself which is too small in cross sectional area as it covers a small amount of the area of the total electrical circuiting, and thus as such the changes in pattern size is considered as negligible. Therefore, similar variations (increase) in the R_th_ for both TC and SSPs are expected by the increase in furnace temperature. Regarding the considerations above, the resistance at the connection point of external wires to the sensor pads persists as the main reason that diverges the reading from SSPs-ave with the assumption that the conventional TC is functioning appropriately. Interestingly, in this arrangement (SBC) the increase in operating temperature contributes positively to wire connection as the compression due to the spring might increase with temperature, which can lead to better connection. This can lead relatively less increase in the resistance than the expected value resulting in relatively less increment in voltage out and corresponding temperature.

Underling the higher temperature reading obtained from the TC during dwelling is the contribution of resistance. As there must be thermal balance during dwelling, given that the furnace is in steady state mode, this should reduce the effect of the thermal inertia. Additionally, a constant difference of lower magnitude (13 °C) between TC and SSPs-ave reading is observed during dwelling (D-TC and D-SSPs ave) compared to the heating segment. These values further decrease during cooling (C-TC and C-SSPs ave) which are obtained only up to the point that failure occurred (Fig. 2). The difference between SSPs ave and TC shows a sharp increase from 8.5 °C to −170 °C due to extremely high Rc (contact loss) leading SSPs’ temperature increase, strangely even when the furnace is in cooling mode.

**Figure 3:**
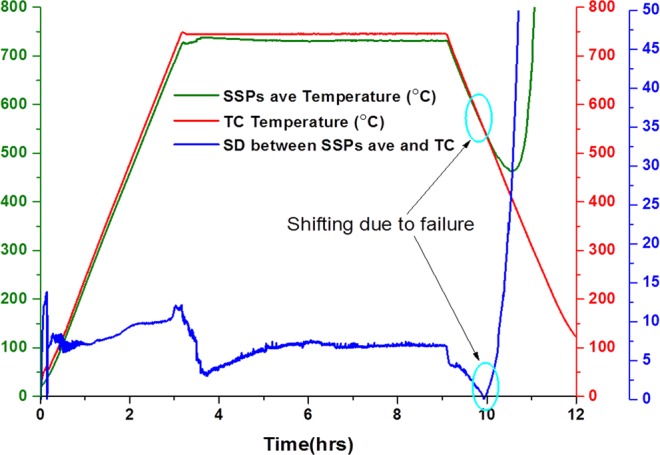
Temperature measurement and standard deviation of Sensor average and TC readings.

Figure 3 shows the standard deviation (SD) of the monitored temperature from both SSPs-ave and TC throughout the experiment. The SD value gradually decreases from heating to cooling segment as SD_heating_ (18 °C) > SD_dwelling_ (13 °C) > SD_cooling_ (8 °C). It is clear from the expression (1) that the thermal inertia of the cell is constant with the assumption that the cell possesses stable material properties during the experiment. However, the contribution of the thermal inertia can alter or even inverted depending on the surrounding environment temperature. As the cell warms up slower and cools down slower (SSPs) than the surrounding atmosphere inside the furnace (TC) during heating and cooling segments, respectively.

Figure 3 also depicts that the SD is not constant during individual segments, as evidenced when it slightly increases by about 3–5 °C during heating segment due to the difference in measured reading, as shown in Fig. 2. The SD is more stable during dwelling compared to the cooling segment and it even goes to even zero during shifting.

Spring expansion from the increase in temperature is observed to have no negative impact to connection security. It is during cooling that the springs start to shrink and lose its spring compression capability (due to creep/plastic deformation/change in elastic modulus occurring from heat treatment) during the decreasing temperature segment, leading to contact loss between pads and external wires. This failure result demonstrates the importance of the materials that could be used in connection purposes in such a high temperature environment. In order to verify this result, another experiment was carried out by applying a very small amount of silver paste to hold the external wires’ connection during the cooling segment and understand the contribution of Rc clearly.

### Results for SBC with applied silver paste

**Figure 4:**
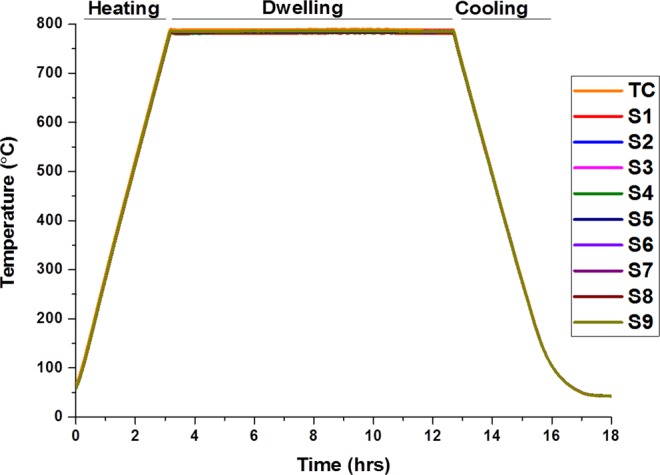
Temperature readings from sensors and TC with SBC.

The obtained temperature readings from SSPs and TC are depicted in Fig. 4. The experimental set up was identical to the setup for the preceding experiment without silver paste. During this experiment a tiny amount of silver paste was used to overcome spring-based issues that occurred during first experiment in the cooling segment and to identify how effectively the SBC connection performs. It was also performed to gain better knowledge about the impact of Rc on the monitored reading. The contact point is now pressure independent due to solid body of cured silver paste. It is clear from the results illustrated in the Fig. 4 that the failure issue is solved. In other words, even though the spring lost its elastic strain restoration the applied silver paste was capable of holding external wires in contact with pads during the experiment. Temperature reading from both the sensor sensing points and TC are in good agreement in both heating and cooling segments as seen in Fig. 4.

**Figure 5:**
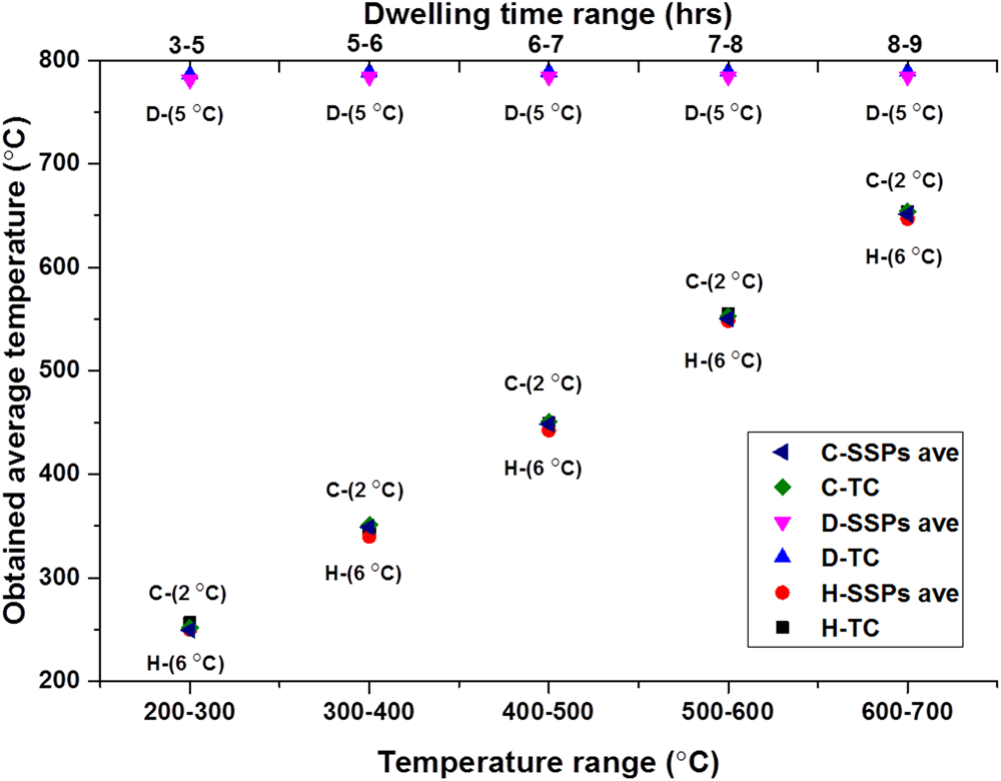
SSPs and TC average temperature from the given temperature range and the differences between them.

As can be seen from Fig. 5, the difference between average temperature readings from TC and SSPs-ave in a given temperature range is constant during each segment with a different value which are 8 °C, 6 °C and 2 °C for heating, dwelling, and cooling segments, respectively.

Different physical structure causes different initial thermoelectric properties, including Rth and Rc, resulting in different corresponding Seebeck potentials and finally different temperature readings. However, the amount of the changes in thermoelectric properties during individual segments seems similar due to similar material properties of the thermoelements resulting in constant difference.

**Figure 6:**
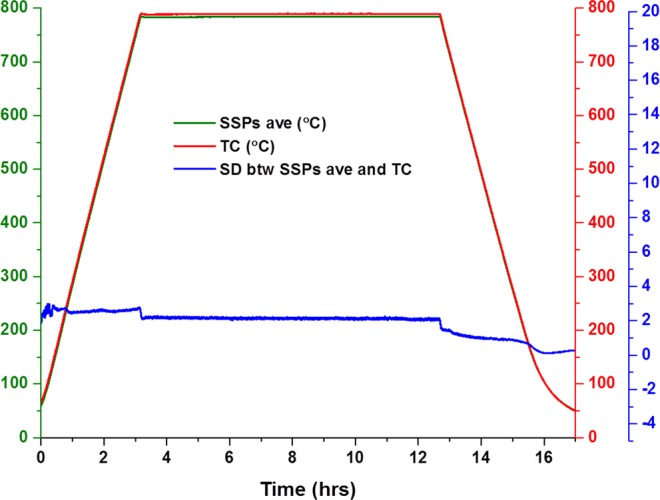
Temperature measurement and standard deviation.

The constant relationship between SSPs-ave and TC average reading is also seen from Fig. 6, showings SSPs-ave and TC readings and their SD changes during the experiment. The SDs was calculated at 2.5 for heating, 2 for dwelling and 1 for cooling segments.

All SD values obtained with this experimental setup are significantly less than the SD values obtained from the experiment without silver paste as depicted in Fig. 5.

As mentioned in section III-A, there are two factors affecting the temperature reading. These are the difference in thermal inertia between the air atmosphere in the furnace and bulk cell, and resistance (Rc and Rth) especially at the contact point for SSPs.

Regarding to the presented results and discussion carried out, the contribution of the factors that generates diversion within the reading from TC and SSPs-ave can be formulised by (4):

4 $${T}_{TC}-{T}_{SSPsave}={T}_{TI}+{T}_{Rth}+{T}_{Rc}$$

where “*T*_*TC*_” is conventional TC reading, “*T*_*SSPs ave*_” is average reading from thin film sensor (S1–S9) and “*T*_*TI*_”, “*T*_*Rth*_”, and “*T*_*Rc*_” are the contributions due to thermal inertia, thermal resistance and contact resistance, respectively. The result depicted in the Figs 5 and 6 proves that the Rc is dominant parameter for diversion of the temperature difference between TC and SSPs-ave during the experiment without silver paste. It is inferred from the fact that thermal inertia effect (which is constant) and the effect due to Rth (which similarly changes for both TC and SSPs) do not contribute the temperature variation between TC and SSPs-ave. In other words, Rc is the only differentiating term among the physical parameters of the implemented sensor which can attribute to the TC diverting the temperature from the expected value. The electrical contact resistance (Rc) of two connected parts can be given by (5)^28^:

5 $$Rc=\{(\rho 1+\rho 2)(1/[4na]+\alpha -1)\}+\rho fs/Ac\,$$

where, the “*ρ*_1_” and “*ρ*_2_” are the specific resistivity, “*n*” is the number and “*a*” is the radius of the contacting asperities, “*ρ*_*f*_” is the resistance of the film between the two parts, “*α*” is the Holm radius, “*s*” is the contaminant, and “*A*_*c*_” is the contact area. The first part of the equation is a constriction effect due to two different mediums while the second part is the effect due to contamination. Differing from the Rth along the conductive materials, the Rc decreases with the increase in operating temperature and applied contact pressure^28^. The connecting materials are being ‘smoothened’ with the elevated temperature resulting in more uniform contacting surface, eventually creating more contact points. The contacting asperities are further increased with the increase in pressure. This proves the presented findings from the experiment without silver paste, as the compressing of the spring is increased with elevated temperature, causing Rc to decrease together with the increase in temperature. Eventually, the increment in the resistance of the whole sensor circuit is relatively low compared to that of the expected value resulting in an increase in temperature difference between SSPs-ave and TC.

### Temperature sensing in operating SOFC

**Figure 7:**
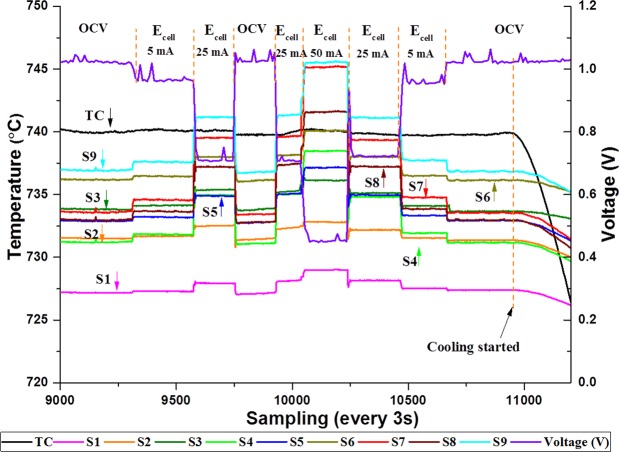
Sensor response to varying loading condition.

The responses of SSPs and TC to variation of cell temperature due to varying loading condition with 20 mL min^−1^ H_2_ and 250 mL min^−1^ N_2_ at 750 °C are shown in Fig. 7. The experimental condition is identical to the condition of experiment where SBC was tested with small silver paste. TC shows the highest temperature reading followed by S9 which shows the highest temperature reading among other SSPs during OCV condition. S1 shows the lowest reading (~728 °C) where the SSPs average temperature and max temperature grading among them are calculated as ~732 °C and ~10 °C, respectively. The OCV of the system is measured as ~1 V.

The monitored temperature readings from the SSPs and TC during OCV condition are relevant to the temperature of their locations and their internal resistance. However when the system is loaded, the monitored temperature variation is due to cell electrochemical activities since the main reaction (H_2_ oxidation reaction) is exothermic. As seen from Fig. 7, the SSPs are sensitive to the temperature changes during loading hence the readings increased with the increase in drawn current whilst the changes monitored from the TC are negligible. Though the maximum temperature reading obtained with S9 is during OCV, S7 shows the maximum increment (~11 °C) when 50 mA current is drawn from the system whilst TC increased about 1 °C only. The SSPs of the implemented sensor provide fast response to the operational variations and are able to return to the original level (repeatability) when the condition is turned to its original state. On the other hand, when the furnace temperature is reduced, as seen at the graph’s last portion (just before 11000s and thereafter), the TC is responding before the SSPs due to the different thermal inertia of the air and the cell as early mentioned. This implies that the TC is more sensitive to the air atmosphere inside the furnace rather than presenting the cell surface temperature. In contrast, the sensor is found to be more sensitive to temperature reading caused by cell activities rather than air temperature inside the furnace.

This study offers a new concept to make applicability of thin film sensors, including stress-related or temperature- related studies easier for SOFC systems. As is seen from the results obtained during loading conditions, the sensor provide higher temporal and spatial resolution compared to the TC. Hence it shows a potential to be an effective tool to provide key information about the operating SOFC with the presented connection method. It is worth noting that the SBC provides significant benefits not only for establishing and maintaining electrical connection between thin film sensor and external wires but also helps smooth bonding of conductive paste materials due to its compression during curing process of paste. However, resistances might be created due to the connection point and thus the different physical structure of the employed sensor is required to be considered carefully for reliable measurement. Consequently, wire attachments with greater electrical and mechanical effectiveness and robustness can be achieved by the contribution of the spring. Therefore, the SBC method can be proposed as a promising candidate to deal with the current-collection related issues due to high resistance at the connection points^8^. However, further investigation is required to identify the feasibility of the techniques for different types of manifolds and for stack-scale systems.

## Methods

### Currently applied method for external wire attachment to a thin film sensor

**Figure 8:**
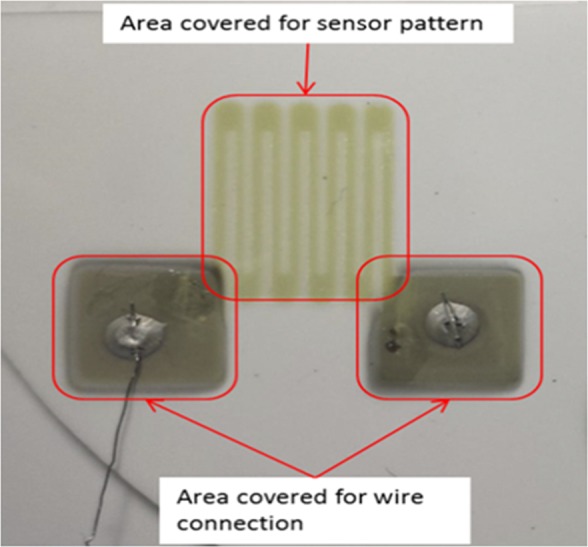
Structured RTD and its connection mechanism^34^.

For those sensors including resistance temperature detectors (RTD), thermistors, resistance strain gauges (RSG), and TCs used in the thin film form, external wire connection plays a key role as a step in the determinant pathway for accurate and constant signal collection^21^. Additionally, the intrusions to signal properties, especially due to aging, parasitic thermal voltages and internal resistance, all have direct correlation with the connection mechanism; furthermore, these mechanisms typically occur slowly and not are hence not easily noticeable, resulting in ‘drift’ and inaccurate sensor readings^29,30^. Therefore, it is paramount to have a mechanically stable and electronically conductive connection. However, conventional wire connection techniques such as welding and soldering are not applicable for certain applications due to their sensitive material characteristics and high working/treatment temperature. As a result, external wires are generally bonded to the thin film sensor by using conductive metal paste as shown by the structure seen in Fig. 8.

Two particular difficulties arise with this connection method, especially when the sensor is used in an SOFC system. Firstly, it is essential to hold the wires in a position of stability and continuous contact, otherwise small external mechanical load from/on the wires (e.g. tension forces or weight) or expansion of the wires themselves, may lead to removal of the wire from the thin film surface, eventually resulting in failure of the connection^21^. Secondly, it can cover a decent amount of area for connection purposes which is larger than the area that is taken up by the sensors themselves, as illustrated by Fig. 8.

However, the surface of the cell is electrochemically and chemically active and a larger active surface area is the key factor to maximise SOFC performance. Furthermore, SOFC should ideally be a gas-tight closed system, which makes external wire connections even more challenging^31^. By considering the SOFC requirements for thin film sensor integration, the following requisites listed:

- minimum disturbance to the nature of operation and its requirements
- minimum space for wire attachment and connection
- no requirement of machining on SOFC cell

In particular, the concerns to overcome the difficulties for wire connection are:

- provisioning for sufficient space;
- extra support to provide constant mechanical strength to hold wires in position;

To fulfil these concurrent demands, a new wire connection method has been developed.

### Spring based connection (SBC) for wire attachment to a thin film sensor

A thin film sensor, which has similar working principles with commercially available TCs in terms of electrical signal acquisition, was recently developed by the authors and was used for temperature sensing purposes in this study. If one end of the two different conductors is joined, there is an electrical potential generated across the unpaired terminals when there is a temperature difference between the joined and free terminals. This is the means by which a conventional TCs measures the temperature of a location, which is also known as Seebeck theory^2,3^.

Therefore, “2*N*” number of thermoelements are required for “*N”* number of sensing points in conventional TCs. However, “*N*^*2*^*”* number of independent sensing points can be obtained with only “*2N*” number of thermoelements (grid structured) with the developed sensor due to its architecture and related algorithm. This is a significant achievement especially pertaining to SOFC systems as there is limited available space.

**Figure 9:**
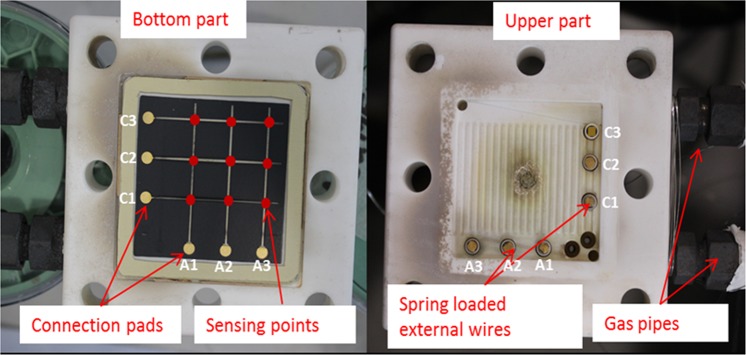
Sensor patterned cell (onto bottom part) and corresponding external wires (onto upper part) configuration.

Figure 9 shows the sensor-integrated cell placed onto the bottom part and the required corresponding spring-loaded external wires were placed into holes built-in on the upper part (see supplementary file section-1 for detailed explanation). The enlarged end of the external alumel (A1–A3) and chromel (C1-C3) wires and the corresponding alumel and chromel connection pads were gold plated to minimise oxidation for better electrical connection.

Nine sensing points (red-dotted) were created by applying a grid structured thin film sensor with three alumel and three chromel which were deposited on the cathode surface by sputtering.

Alumel and chromel thermoelements were selected to form K-type TC junctions due to its wider working temperature range up to 1200 °C and lower material cost^32,33^. The sensor thermoelements were encased with an alumina protecting layer to prevent any detrimental harm from the working environment. Additionally, the deposited alumina and gold thin film at the connection pads blocks the penetration of the applied connection paste material into the porous cathode (in the case of using conductive Ag paste for wire bonding) as the surface of the sputtered film is more dense than the surface of the cathode electrode. The sensor provides the local surface temperature individually at each junction (sensing points) on the cathode.

**Figure 10:**
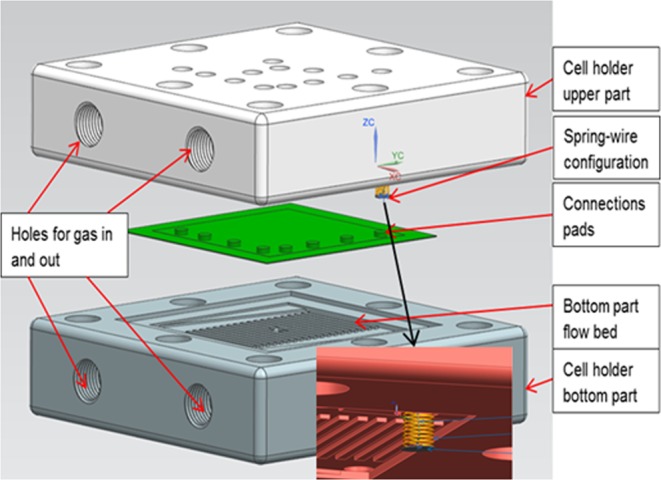
Schematic drawing of the assembly of cell holder with cell and spring integration.

Figure 10 shows the schematic of the assembly of the manifolds’ upper and lower parts with integrated spring, wires, and cell configuration. A spring (stainless steel) is placed within the built-in holes contained in the upper part of the manifold for sensor integration, to exert a normal load at the connection point, and the external wires are sent from outside the upper part of the cell holder to the interior (through the aforementioned spring located in the hole), to contact with pads at the connection point.

The key point here is that the mask used to generate the desired shape of the sensor and connection pads is aligned with the holes where external wires come through. The pads were located at edge of the cell active area (shown in Fig. 10) minimising disturbance to cell active surface.

Diameter of the enlarged edge (3 mm in Dia.) of the wires is larger than the diameter of the spring (2.95 mm in Dia.) and less than the holes’ diameter (4 mm in Dia.). With this configuration, the spring applies a compressing load to the connection depending on the free length and the compression rate of the spring employed. When the two parts of the cell holder are joined the free length of the spring is compressed to the channel wall level which is where the cell surface is in contact. The aim and intent with this connection configuration is to limit disturbance to the operating conditions (including flow distribution, gas penetration and electrochemical reactions) to a minimum, whilst simultaneously keeping wires in continuous contact with the thin film sensor pads.

## Experimental procedure

Two different set ups were prepared and applied for the experiment: one with silver paste and another one without silver paste as a conductive connection material. In the first case, external wires were directly attached without any paste, between the enlarged end of the wires and connection pads, and for the second instance external wires were attached with a very small amount of silver paste and system is loaded. For both cases, the SBC technique was applied.

The thin film sensor-integrated cell was placed in the cell holders’ bottom part while the external wires’ connection set up was placed onto upper part. The cell was sandwiched by two gaskets. The gaskets, which provide the mechanical sealing mechanism, were placed in the upper and lower parts. The material used for sealing was Thermiculite 866 (Flexitallic Ltd.), cut as a hollow square, with outer dimensions approximately 52 mm × 52 mm, and inner dimensions of 40 mm × 40 mm (Fig. 9).

A sensor-integrated compact test kit is completed by assembling the upper and bottom part of the manifold. Then the completed test kit was placed within a high temperature box furnace. No gas apart from nitrogen was supplied during the heating process and throughout the experiment during investigating the effectiveness of external wire connection. After the wire connection is achieved, temperature behaviour of the cell with varying loading condition is also investigated under real SOFC condition. There was a commercial k-type TC also placed closely (2–3 mm above from the surface of the cell) to compare with the readings obtained from sensor sensing points. Once the assembly was completed, the furnace was heated to 750 °C with a 2 °C min^−1^ heating rate and cooled down to room temperature after about 5 hours dwelling at 750 °C with the same cooling rate for the first set up. During the second set up furnace temperature increased to 800 °C with 2 °C min^−1^ and dwelled 5–6 hours then cooled down to room temperature. After the external wire electronical connection is confirmed the cell is heated to 750 °C again and its temperature behaviour is monitored under loading SOFC condition by supplying H_2_ together with N_2_ heating. Before the system is loaded, the anode reduction process was completed. Dedicated LabVIEW software was created in-house to perform the duties of collecting and recording the data throughout the experiment.

## Conclusions

The performance of the reliability of signal acquisition via SBC with a new manifold was investigated. Electrical signals (as temperature measurement from nine different points) were acquired by applying a recently developed thin film sensor. External wires through the cell holder’s upper part and the enlarged edge of the wires were attached to sputtered thin film sensor pads to complete electrical connection. For the first experiment without any silver paste at the connection point, failure was observed during the cooling segment due to spring-based issues, namely the loss of proportional and elastic behaviour (change in elastic modulus) from exceeding the temperature ceiling of the spring. Secondly, an experiment was run with a small amount of silver paste to overcome this issue. Using a high temperature spring that can maintain its compression characteristics during heating and cooling is alternate pathway to solve the failure problem. Analysing the result of those two experiments, it is found that the spring-based connection for the SOFC technology is an efficient method for signal collection, with less affects from the normal operating conditions. The authors would like to point out that there is a high possibility to maintain the sufficient electrical connection without using silver paste at the connection points, but requires the use of a high temperature spring. In addition, the SBC technique is considered as an effective means for other applications that require electrical signal transmission and control, but present limited availability to sensor implementation. Furthermore, it leads to scale up of the viability of thin film sensor technology, given that the wire connection has been standing as a significant obstacle thus far, and aid in the group activities for developing the temperature sensor array for studies for failure detection and mitigation by deep learning, as well as incorporation into model-based predictive control.

## Supplementary information

Supplementary Materials
