# Supplementary material for: Spring Based Connection of External Wires to a Thin Film Temperature Sensor Integrated Inside a Solid Oxide Fuel Cell
Source: Sci Rep. 2019 Feb 15;9:2161. doi: 10.1038/s41598-019-39518-2 (PMC6377679; doi:10.1038/s41598-019-39518-2)
Supplement: Supplementary file 1 — Supplementary Materials [file 41598_2019_39518_MOESM1_ESM.pdf]

Supplementary Materials

Spring Based Connection of External Wires to a Thin Film Temperature Sensor Integrated Inside a Solid Oxide Fuel Cell

Erdogan Guk, Vijay Venkatesan, Yunus Sayan, Lisa Jackson, Jung-Sik Kim[1]

[1] Corresponding author
Email: j.kim@lboro.ac.uk ; Tel: +44 (0)1509 227 219 Fax: +44 (0)1509 227 275

1. Description of the bespoke SOFC manifolds:

A multifunctional lab-scale (house-made) cell holder (manifold), comprising flow channels (bed), holes for fuel and oxidant inlets and outlets, and orifices for wire connection, was designed as shown in Fig. S1 (bottom part) and Fig. S2 (upper part). Macor as a machinable ceramic was used as the manifold material. Since its coefficient of thermal expansion is compatible with most metals and sealing materials, it also provides great electrical insulation even at high temperature up to 1000 °C [1]. The holes in the centre of both lower (Fig. S1) and upper parts (Fig. S2) facilitate wire connections for the current collection while the holes on the side of the upper part are specific considerations for wire attachments for the sensor electrical connection (Fig. S2).

Manifold design and its functionality is an important factor that helps fuel and oxidant to be uniformly distributed, yielding positive impact on SOFC performance [2, 3]. The designed manifold consists of two parts: (i) an upper part where the oxidant is supplied (and contains an exit path), and also has holes for wire attachments for thin film sensors' electrical circuit connection, and (ii) a lower part in which the fuel is supplied, and has only a single hole for electrical connection for the current collection mechanism employed.

The bottom part (anode side) of the cell holder, as shown in Fig. S1, has two main sections: (i) a flow bed (blue coloured) including a triangular enlarging inlet, parallel flow channels in the middle and a flow outlet which has a similar structure with the flow inlet; (ii) a cell bed (green coloured), where the cell electrolyte sits, along with sealing materials.

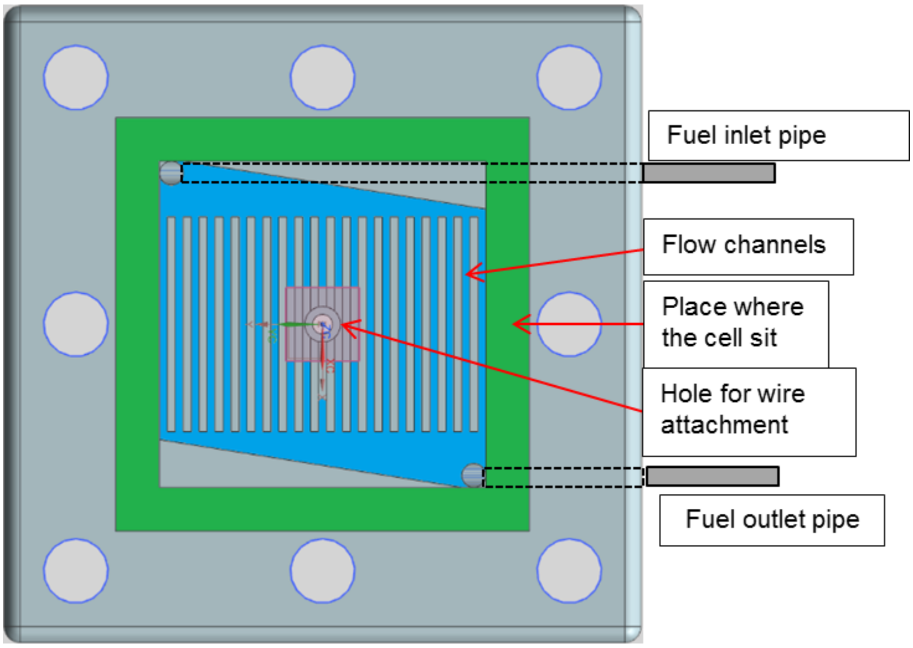

Fig. S1. Schematic view of bottom (anode side) of the cell holder

Chemical sealing or gaskets are to be placed on the cell bed before the cell in order to sandwich the electrolyte flush with the upper and lower holder parts, to prevent fuel leakage from anode side to cathode side (or vice versa).

In this case, the cell will be sandwiched between two gaskets to be compressed safely. The eight bores on the outer side of the bottom part are for fasteners (threaded rod/nut type) to connect the two parts of the cell holder with required tightness. This can be modified to apportion the appropriate number and type of fasteners to ensure requisite pressure/screw torque if mechanical gaskets are employed.

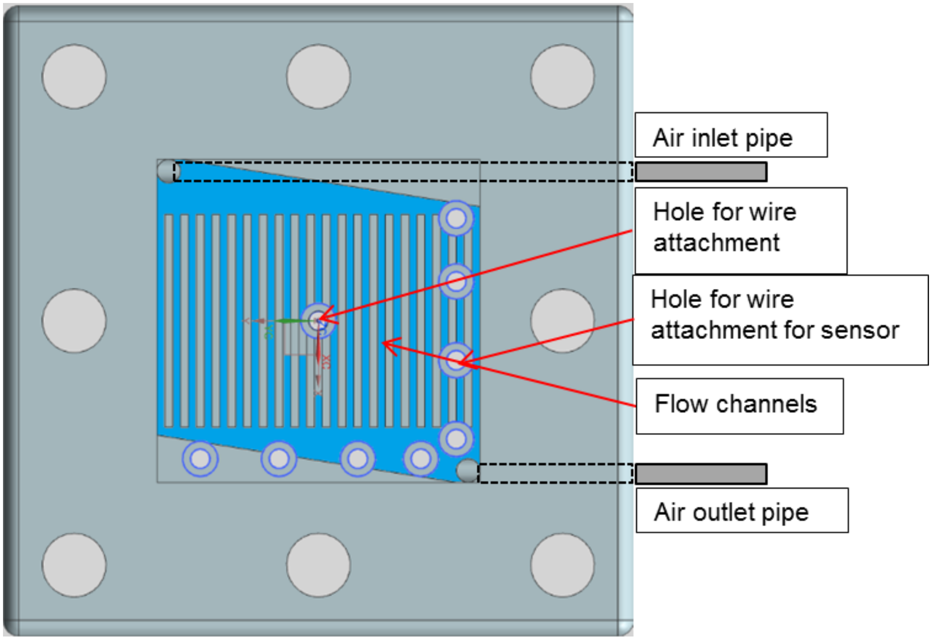

Fig. S2. Schematic view of upper (cathode side) of the cell holder

The upper part (cathode side) of the cell holder as shown in Fig. S2 also consists of two main sections, namely a flow bed having the same geometric configuration with the bottom part as per the previous paragraph. The hole in the centre is to be used for cell current collection and the holes on the outer side of the flow bed are to be used for sensors' electrical connection. This is accompanied by a cell bed designed to accommodate the electrolyte of the cell. This particular manifold is specifically designed for a 50mm × 50mm sized planar cell with an allowance of different configurations such as electrolyte, anode or cathode supported cell.

References

1. Corning Inc. *Macor data sheet*. (2001).

2. Argyropoulos, P., Scott, K. & Taama, W. M. Modeling Flow Distribution for Internally Manifolded Direct Methanol Fuel Cell Stacks. *Chem. Eng. Technol.* **23,** 985–995 (2000).

3. Metzger, P., Friedrich, K. a, Muller-Steinhagen, H. & Schiller, G. SOFC characteristics along the flow path. *Solid State Ionics Notes* **177,** 2045–2051 (2006).
